# Fast and flu-rious: How to prevent and treat emerging influenza A viruses

**DOI:** 10.1371/journal.ppat.1013135

**Published:** 2025-05-16

**Authors:** Isabel Sesifredo, Íris Luz Batalha, Maria João Amorim

**Affiliations:** 1 Cell Biology of Viral Infection Lab, Universidade Católica Portuguesa, Católica Medical School, Católica Biomedical Research Centre, Lisbon, Portugal; 2 Department of Life Sciences, University of Bath, Claverton Down, Bath, United Kingdom; University of Wisconsin-Madison, UNITED STATES OF AMERICA

## Influenza A: The zoonotic virus behind the flu

**Fig 1:**
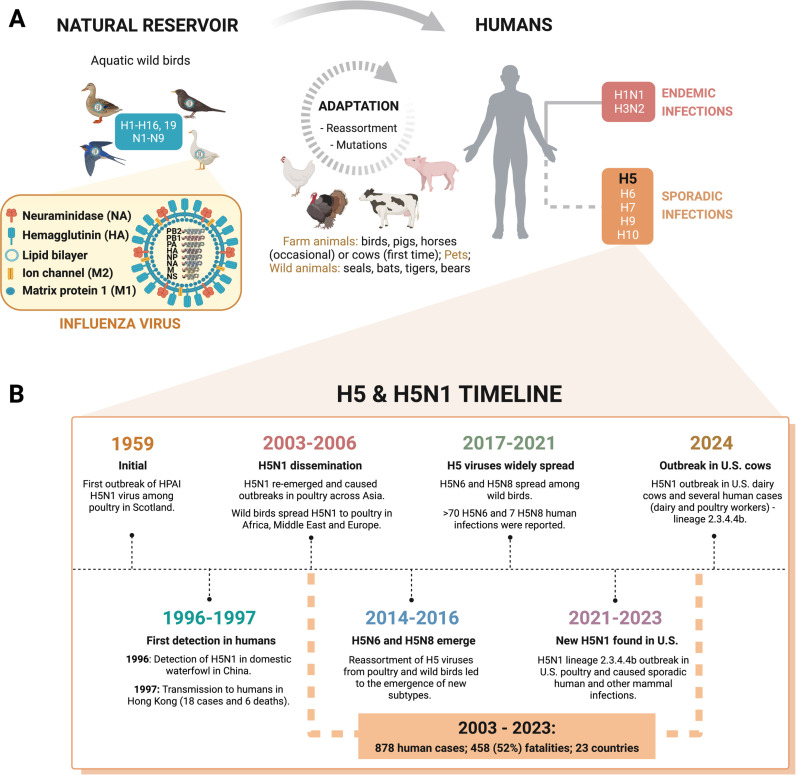
Influenza A virus diversity & host spillover. **(A)** IAV transmission. IAV jumps from its natural reservoir to different animals, including farmed animals, undergoing adaptation to this new biological environment. The virus can infect humans sporadically or acquire human-to-human transmission traits and become endemic such as the currently circulating subtypes H1N1 and H3N2 that cause yearly epidemics. **(B)** H5 and H5N1 timeline. HPAI H5N1’s first outbreak was reported in 1959 and first detected in humans in 1997, leading to 6 deaths. Since then, H5N1 and other emergent subtypes (e.g., H5N6 and H5N8) have caused several outbreaks with many human fatalities. Recently, the new H5N1 clade 2.3.4.4b caused an outbreak in U.S. dairy cows, subsequently transmitted to humans (dairy farm workers), threatening human health. Created with Biorender.

Influenza (flu) is an acute and highly contagious respiratory disease that triggers annual outbreaks and sometimes deadly pandemics [1]. It is caused by two of the nine genera of the *Orthomyxoviridae* family, which are segmented negative-sense single-stranded RNA viruses, namely influenza A (IAV) and influenza B (IBV) viruses [2]. However, IAV poses an additional pandemic risk due to its wide host range and ability to occasionally spread from its reservoir (aquatic wild birds) to different species (Fig 1A) [3]. In the reservoir, the antigenic proteins decorating the viral envelope, haemagglutinin (HA or H) and neuraminidase (NA or N), are highly diverse, comprising seventeen HA (1–16, 19) and nine NA (1–9) subtypes [4]. Each subtype is subdivided into clades based on HA similarities and each clade can accommodate several genotypes based on differences in all segments but HA [5].

When a virus jumps to a new species, it must adapt to its biological environment, which often happens silently through stepwise mutation events and/or reassortment (genetic mixing between segmented genomes) [3]. In particular, the circulation of the highly pathogenic avian influenza (HPAI) H5N1 virus, capable of causing systemic disease amongst animals, has been a growing concern, especially in animals in close contact with humans as it increases the probability of inter-species adaptation (Fig 1B) [6]. While outbreaks in poultry have been documented since 1959 and in humans since 1997 [6], the virus has recently expanded its reach to infect a wider range of domesticated and wild animals, including cats, dogs, polar bears, sea lions, and, most recently, cattle [7].

Although H5N1 infections generally lack key adaptations to sustain in mammals (i.e., mammal-to-mammal transmission), there have been exceptions, including marine mammals (South America), fur farming animals (Europe), and dairy cattle (U.S.) with intra-species transmission reported [8]. Recent cattle-to-cattle transmission has been proposed to occur through milk and milking procedures, subsequently leading to human spillover events that resulted in 1 death among 70 confirmed cases [8,9]. The mild severity may be related to the attenuation of this specific genotype or the route of infection (i.e., through the eye) [8], given that the overall fatality rate caused by H5N1 infections is 52% (Fig 1B) [6].

The finding of multiple, antigenically different H5N1 HA clades in over 50 different animal species makes it impossible to predict which clades will be transmitted and maintained in humans, if any [10]. These factors and the virus’ pandemic potential underscore the critical need for effective outbreak control, tight surveillance, impact mitigation, and human transmission prevention, for preparedness in case of H5 establishes in humans [11].

## Flu shots: Great, but still not perfect—Here’s why

The development of a universal influenza vaccine has not been possible because of the high diversity of antigenic regions within the HA and NA proteins of influenza viruses. As a result, current vaccines target specific IAV and IBV strains, and do not fully encompass the antigenic characteristics of all circulating and zoonotic strains [12]. Candidate vaccine viruses (CVVs) against specific strains are selected biannually, aided by the Global Influenza Surveillance and Response System [12]. This requires mapping the prevalent circulating strains, quantifying the neutralisation potential of vaccines, and measuring HA titres on prevalent strains [13]. These efforts have greatly reduced influenza mortality and morbidity. However, as vaccine efficacy varies, occasional updates, dictated by a 4-fold or more decrease in neutralisation or HA inhibition titres, are necessary [14].

CVVs for all circulating H5 subtypes have been identified, which is crucial to shorten the response to zoonotic outbreaks [15]. Selected strains for vaccine production for the prevalent H5N1 clade in circulation (2.3.4.4b) confer protection to many circulating genotypes (B3.13, D1.1, and D1.2) within this clade, and even to different clades or subtypes [8]. For example, early H5N1 clade 1 vaccines provide some, although less, protection against clade 2.3.4.4b [16]; and older people are partially immune to H5N1 because of exposure to seasonal viruses (H1N1 and H2N2) during childhood [8]. H5 viruses (contrary to seasonal H1 or H3) primarily infect naïve hosts, facing less and slower immune-driven selection pressure, which can reduce the risk of mismatch [17]. Given these circumstances, vaccine stockpiling is a key aspect of pandemic preparedness. Nevertheless, there are some challenges to overcome. For example, H5N1 vaccines have demonstrated poor immunogenicity in clinical trials, even when adjuvants were included in formulations using inactivated virions [18].

Influenza vaccines were traditionally produced in eggs, but cell-based production has become widespread since 2019/2020 [19]. Both egg- and cell-based vaccines require cumbersome virus purification and inactivation processes [19]. mRNA-lipid nanoparticle (LNP) vaccines offer the potential for rapid production and lower costs but still face challenges related to transportation and storage [13]. mRNA-LNP vaccines were well tolerated and elicited robust humoral immune responses in a Phase I clinical trial against potentially pandemic H10N8 and H7N9 viruses [20]. The mRNA-LNP vaccines under preclinical testing against H5N1 clade 2.3.4.4b viruses may bypass the lack of immunogenicity mentioned above [21]. Given these factors, vaccines should be seen as one component of a broader pandemic preparedness strategy.

## Influenza’s intrinsic power: How it keeps spreading

Despite the existence of available treatments and effective vaccines, several factors contribute to the perpetuation of IAV:

i) IAV replication is carried out by an RNA-dependent RNA polymerase (RdRp) that lacks proofreading capacity, giving rise to errors during replication [22]. These errors can manifest in any viral protein, resulting in a diverse array of viral mutants or variants that can present evolutionary advantages in certain conditions (e.g., infecting different host species, resisting antiviral treatments, evading immune pressure). When these mutations are present in HA or NA, it is known as antigenic drift [23]. These mutants may escape pre-existing immunity, including neutralising antibodies, and render antivirals ineffective, leading to reinfection.
ii) The segmented nature of the IAV genome facilitates the exchange of genetic material between different strains that co-infect the same cell (i.e., reassortment). This process accelerates viral evolution, allowing IAV to acquire genetic material already adapted to a particular environment [22]. This mechanism has been instrumental in the emergence of IAV strains responsible for the 1957, 1968, and 2009 pandemics, as it confers a substantial advantage during host spillover events [22].
iii) Many respiratory viruses, including IAV, are contagious before individuals develop symptoms. Additionally, infected individuals with mild symptoms may fail to isolate themselves, contributing to further viral spread [24].
iv) Vaccines are the primary preventive measure against IAV and IBV infections, and are highly effective in reducing the risk of severe illness [12]. But, occasionally, they fail to provide complete protection due to mismatches with circulating strains, low vaccine coverage (i.e., restricted to high-risk groups, such as immunocompromised or immunosenescent individuals), or individual variability (e.g., age, weight, sex, or immune status) [25].

## Antiviral drugs: The weapons to mitigate the impact of IAV

Influenza infections can be combated through two primary approaches: vaccination as a preventive measure and antiviral therapies for prophylactic and therapeutic purposes [26]. So far, the U.S. Food and Drug Administration (FDA) has approved three main classes of antiviral drugs (Table 1): M2 ion channel inhibitors (amantadine and rimantadine), no longer recommended for monotherapy due to drug resistance, NA inhibitors (zanamivir, oseltamivir phosphate, and peramivir), and viral polymerase inhibitors (baloxavir marboxil) [27].

**Table 1:** Clinically approved antiviral drugs against influenza viruses.

Drug	Viral target	Mode of action	Virus	Administration	Mutations associated with resistance	Age treatment permitted	Age prophylaxis permitted	Approval country/date	Ref
No longer recommended									
Amantadine	M2 ion channel	Inhibits viral uncoating and replication	IAV	Oral	S31N, V27A, L26F, A30V, G34E, and L38F	–	–	1966	[25,30]
Rimantadine								1993	
Recommended									
Zanamivir	NA active site	Impairs viral release	IAV and IBV	Inhalation	H274Y, R292K, N294S, E119A/G/D, Q136K, and I222R/K/V	≥7 years	≥5 years	1999	[25,31]
Oseltamivir				Oral		≥2 weeks	≥1 year		
Peramivir				Intravenous		≥2 years	–	2014	
Baloxavir marboxil	PA	Blocks viral transcription	IAV and IBV, including NAI-resistant strains	Oral	I38T/F/M, E23G/K, A37T, and E199G	≥5 years		2018	[32]
Approved in specific countries									
Arbidol	HA Stalk	Inhibits viral entry	IAV and IBV	Oral	K51N, K117R, Q27N, and Q42H	≥5 years		Russia, 1993 China, 2006	[33]
Favipiravir	RdRp	Inhibits transcription and replication	IAV, IBV, and ICV	Oral	K229R	Adults		Japan, 2014	[34]
Laninamivir	NA active site	Inhibits NA activity	IAV and IBV	Inhalation	D197E and E119G	≥5 years		Japan, 2010	[35]
Ingavirin	NP	Blocks NP transport to the nucleus	IAV and IBV	Oral	–	13–17 years		Russia, 2009	[36]

M2: Matrix-2 protein (viral envelope).

NA: Neuraminidase (viral envelope).

PA: Polymerase acidic protein (viral polymerase subunit).

HA: Haemagglutinin (viral envelope).

NP: Nucleoprotein (linked to the production of viral ribonucleoprotein complexes).

Recent *in vitro* studies show that H5N1 clade 2.3.4.4b viruses are susceptible to NA and polymerase inhibitors, and even to M2 ion channel-blockers [28]. Additionally, the polymerase inhibitors baloxavir marboxil and favipiravir were also reported to be highly effective against this clade of H5N1 both *in vitro* and *in vivo* [29].

Additional drugs are approved in specific countries (Table 1), and several are currently in clinical trials (Table 2). However, with an ever-growing increase in drug resistance, new antiviral drugs and strategies are in high demand [25].

**Table 2:** Small molecules, peptides, and monoclonal antibodies against influenza viruses in clinical trials.

Drug	Mode of action	Administration	Adverse effect(s)	Status^*^	Ref
Small molecules and peptides					
Atorvastatin	Inhibits lipid-droplet formation in the viral envelope	Oral	–	Phase II	[37]
Danirixin	Blocks neutrophil chemotaxis in inflammation		Headache, nausea, and dizziness	Phase II	[38]
Nitazoxanide	Affects HA maturation		Headache	Phase III	[39]
Enisamium iodide	Inhibits RdRp activity		–	Phase II	[40]
Pimodivir	Inhibits PB2		Diarrhoea	Phase III	[41]
Ribavirin	Inhibits RdRp activity		Nausea, vomits, and diarrhoea	Phase II^**^	[42]
DAS181	Mimics sialic acid to prevent viral entry	Inhalation	Elevated alkaline phosphatase	Phase III	[43]
Flufirvitide-3	Broad spectrum entry inhibitor		–	Phase II	[44]
Monoclonal antibodies					
MHAA4549A	Targets highly conserved HA epitopes and neutralises virus	Intravenous	Headache	Phase II	[45]
MEDI8852	Broad-spectrum neutralising activity		Headache, hypoglycaemia, and bronchitis	Phase II	[46]
VIS410			Mild diarrhoea	Phase II	[47]
CR6261	Prevents fusion of cellular and viral membranes in endosomes		–	Phase II^**^	[48]
CR8020	Prevents fusion and viral entry			Phase II	[49]
TCN-032	Inhibits M2 protein			Phase II	[50]

HA: Haemagglutinin (viral envelope).

RdRp: RNA-dependent RNA polymerase (viral polymerase).

NP: Nucleoprotein (linked to the synthesis of viral ribonucleoprotein complexes).

PB2: Polymerase basic 2 protein (viral polymerase subunit).

*Status of each drug according to the EU Clinical Trials Register.

**Tested in clinical trials in combination with other drugs.

## The future of antiviral treatment is taking shape

Advances in several key areas are needed for antiviral development to leap forward. Targeting essential viral proteins, cellular pathways exploited by viruses, or the modulation of host immune responses may continue to provide effective therapies. However, understanding new layers in viral–host interactions’ biology may underpin completely novel (and needed) avenues for therapeutic intervention. For example, recent research has shown that cells compartmentalise selected reactions and factors into dynamic, membraneless structures called biomolecular condensates (also known as viral inclusions or factories), which viruses exploit for their benefit [51]. In this regard, it has become apparent that infection often relies on specific material properties, suggesting that targeting the material properties of these compartments may provide a new antiviral approach [52]. For IAV and respiratory syncytial virus, liquidity of viral inclusions is essential, and hardening them was shown to inhibit viral replication *in vivo* [53].

Another issue is that current antiviral drug screening relies heavily on cell lines that don’t accurately reflect the complexity of human tissues. In addition, few preclinical animal models replicate both symptoms and transmission (e.g., chicken and ferret), and all require viral adaptation to some extent (e.g., mice), which often leads to failed clinical translation [54]. Moving towards more human-relevant models, such as those incorporating immune barriers and microbiomes, is vital. While promising technologies like organ-on-a-chip exist, their widespread use is still limited [55].

On the other hand, the emergence of advanced artificial intelligence and machine learning tools, exemplified by AlphaFold, is becoming a game-changer in antiviral drug discovery [56]. These tools enable the exploration of vast chemical spaces, facilitating the design of molecules that can disrupt critical virus–host or virus–virus interactions. Specifically, these tools can be used to: predict antiviral resistance using decision trees and neuronal networks [57]; assess candidate target functions based on known antiviral drugs (e.g., AVPIden) [58]; identify druggable targets for therapeutic intervention; and optimise virtual screening, reducing drug design time and cost [59].

Finally, the field of nanomedicine, spurred by the Covid-19 pandemic, is also one to watch. Nanoparticles can deliver antiviral agents specifically to infected sites, thereby reducing toxicity, or directly eliciting an antiviral response. Metal and metal-oxide nanoparticles, such as gold, silver, and zinc oxide, exhibit broad antimicrobial properties through various mechanisms, including cell membrane disruption and reactive oxygen species generation [60]. For example, ZrO_2_ nanoparticles have reduced IAV replication and inflammation in mice infected with H5N1 [61]. Furthermore, nanoparticles can be combined with existing antiviral therapies to enhance their effect. For instance, selenium nanoparticles with amantadine have been shown to reduce viral activity and inhibit apoptosis in H1N1-infected cells [62].

**Fig 2:**
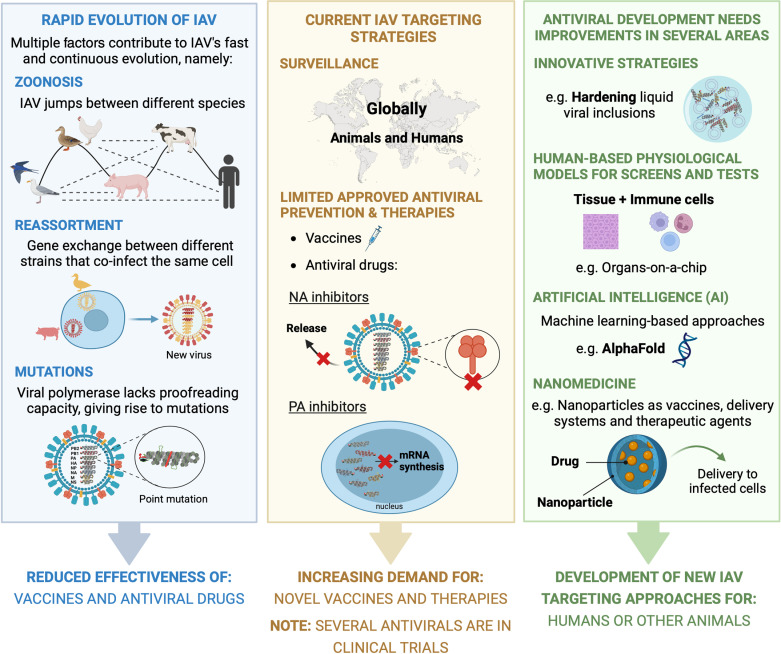
Take home message. (Blue): Rapid evolution of IAV. It relies on a vast viral repertoire widespread through many species, the ability to reassort, and a high mutation rate (the latter two allow adaptations to new biology). All these factors render vaccines and antivirals ineffective. (Yellow): Current IAV targeting strategies. Established global surveillance among animals and humans is key for selecting effective vaccines against circulating IAV strains, and preventing future outbreaks. Today, prevention and therapeutic solutions are limited. The emergence of drug-resistant variants demands the development of new therapeutic alternatives. (Green): Antiviral development needs improvements in several areas. Identification of new antiviral strategies targeting previously unknown viral-host biology (e.g., viral inclusions), better human-based platforms for screening, testing, and validating drugs (e.g., organs-on-a-chip), implementation of technological approaches that bypass one-on-one testing (e.g., AlphaFold) in drug discovery and drug resistance forecasting, and refined delivery systems (e.g., nanoparticles). Created with Biorender.

Overall, despite fast viral evolution and the limitations of current therapies, rapid development in many key enabling technologies is shaping, which are expected to deliver innovative solutions for the prevention, diagnosis, and treatment of viral infections, including influenza (summarised in Fig 2).
